# The European Hare (*Lepus europaeus*): A Picky Herbivore Searching for Plant Parts Rich in Fat

**DOI:** 10.1371/journal.pone.0134278

**Published:** 2015-07-31

**Authors:** Stéphanie C. Schai-Braun, Thomas S. Reichlin, Thomas Ruf, Erich Klansek, Frieda Tataruch, Walter Arnold, Klaus Hackländer

**Affiliations:** 1 Department of Integrative Biology and Biodiversity Research, Institute of Wildlife Biology and Game Management, University of Natural Resources and Life Sciences, Vienna, Vienna, Austria; 2 Division of Animal Welfare, Vetsuisse Faculty, University of Bern, Bern, Switzerland; 3 Department of Integrative Biology and Evolution, Research Institute of Wildlife Ecology, University of Veterinary Medicine, Vienna, Vienna, Austria; University of Lleida, SPAIN

## Abstract

European hares of both sexes rely on fat reserves, particularly during the reproduc-tive season. Therefore, hares should select dietary plants rich in fat and energy. However, hares also require essential polyunsaturated fatty acids (PUFA) such as linoleic acid (LA) and alpha-linolenic acid (ALA) to reproduce and survive. Although hares are able to absorb PUFA selectively in their gastrointestinal tract, it is unknown whether this mechanism is sufficient to guarantee PUFA supply. Thus, diet selection may involve a trade-off between a preference for energy versus a preference for crucial nutrients, namely PUFA. We compared plant and nutrient availability and use by hares in an arable landscape in Austria over three years. We found that European hares selected their diet for high energy content (crude fat and crude protein), and avoided crude fibre. There was no evidence of a preference for plants rich in LA and ALA. We conclude that fat is the limiting resource for this herbivorous mammal, whereas levels of LA and ALA in forage are sufficiently high to meet daily requirements, especially since their uptake is enhanced by physiological mechanisms. Animals selected several plant taxa all year round, and preferences did not simply correlate with crude fat content. Hence, European hares might not only select for plant taxa rich in fat, but also for high-fat parts of preferred plant taxa. As hares preferred weeds/grasses and various crop types while avoiding cereals, we suggest that promoting heterogeneous habitats with high crop diversity and set-asides may help stop the decline of European hares throughout Europe.

## Introduction

The European hare is the smallest mammalian species in Europe dwelling above ground or without shelter throughout the year. Consequently, this herbivore has higher energetic costs for growth, thermoregulation, parental care, etc. than mammals of the same size that use burrows, nests or dens. In line with this, European hares produce precocial, rapidly growing young, and females provide milk with a high fat content of more than 20% dry matter (DM) [1–2]. The milk fat for the first litters in spring derives predominantly from fat reserves which were built up during winter [3–5]. Until summer, fat reserves are depleted [3, 6] and in autumn, lactating females are forced to increase energy intake rates [6–7]. In the course of the prolonged breeding season which lasts from January until October [3], female hares are thus capital breeders in spring and income breeders later. Irrespective of seasonal differences, it seems clear that reproduction in hares requires a high intake of dietary fat.

The crude fat content in the food plants of herbivorous mammals is typically less than 3.5% [8]. Thus, dietary crude fat may represent a limited resource for this group. In European hares, the mean proportion of crude fat content in the gastrointestinal tract content is more than 30% higher than expected for herbivorous animals (4.6% [5]). Moreover, mammals must obtain essential polyunsaturated fatty acids (PUFA), namely linoleic acid (LA C18:2 n-6) and alpha-linolenic acid (ALA C18:3 n-3) from their diet, because they lack the enzymes necessary for the synthesis of these fatty acids (FA). Increased intake of PUFA, in particular LA, increases the reproductive performance of European hares: dietary PUFA supplementation significantly increased the number of leverets born and successfully weaned [9]. In line with this, Popescu et al. found a significantly higher proportion of LA in the gastrointestinal tract content of lactating females than in that of non-lactating females [5].

Dietary PUFA are relevant for a number of physiological traits. A high PUFA content in muscle membrane enhances muscle activity [10–14]. Valencak et al. found a high degree of muscle membrane unsaturation in the European hare and linked this to the extraordinarily high maximum running speed of this species [15] (more than 70 km/h [3]). Moreover, high PUFA content in membranes helps to maintain functionality at low temperatures [16]. Therefore, Valencak et al. assumed that the high PUFA content found in skeletal muscle in winter reflects thermoregulatory adjustments, e.g. regional heterothermy, to the severe climatic conditions in Central European winters [15].

European hares should, on the one hand, feed selectively on plants rich in fat [1–2], but on the other hand, they should feed selectively on plants rich in PUFA, such as LA [5]. This might lead to a trade-off between maximising energy intake and maximising the intake of specific plant compounds, in this case PUFA. Smith et al. found that hares did not prefer habitats supporting plants of high nutritional quality [17] (crude fat, crude protein or energy, see also [18]). This could be due to the fact that hares feed selectively on specific crops, weeds and grasses [19–21] avoiding most of the available plant biomass [22]. This selectivity might indicate preferences for specific nutrients. However, at least with respect to PUFA intake, hares may also rely entirely on physiological mechanisms to ensure sufficient supply. Popescu et al. found that European hares selectively absorb PUFA in the gastrointestinal tract, excreting faeces that are highly depleted in PUFA and enriched in saturated FA [5]. It remains unclear, however, whether this mechanism alone is sufficient to guarantee adequate PUFA intake.

The goal of this study was to investigate nutrient and forage preference in the European hare throughout the year in an agricultural area. In particular, we focussed on the botanical and chemical composition of forage taken by hares, and examined the potential influence of age, sex and season on the food selected. It seemed important to differentiate between seasons, because the chemical composition of the available plants (e.g. the proportions of various nutrients and FA) differs between different plant taxa, plant parts, and developmental stages of plants in the course of the year [23–26] and because the nutritional needs of female hares may change throughout the long breeding season.

We asked whether hares select food in order to meet their needs for dietary fat or, specifically, their needs for PUFA. Our reasoning was as follows: (1) if selective uptake of PUFA in the gastrointestinal tract can supply them with sufficient essential FA, we hypothesize that European hares will choose a diet with high energy content, preferring plants and plant parts rich in fat. This may even lead to an apparent avoidance of plants rich in certain PUFA, unless they also have high energy content. (2) Alternatively, if digestive mechanisms that enhance PUFA uptake are insufficient, we hypothesize that hares should prefer plant taxa rich in PUFA, namely LA and ALA. (3) Under both the above scenarios, we hypothesize that dietary preferences vary according to season, individual age and sex. We tested the hypotheses by analysing both the botanical and chemical composition of hare stomach contents, and comparing the values with those of the food plants available in four seasons for three years in an arable area in Austria. We aim to use knowledge of dietary preferences to make recommendations for the conservation of the hare through targeted habitat management.

## Materials and Methods

### Study area

The study was conducted in the Marchfeld area in Lower Austria (48°11’N, 16°42’E) during the years 2003–2005. The study area consisted of four hunting grounds of 2173 ha arable land with an average field size of 1.7 (± 0.02 SE) ha. Cereals (28%), predominantly winter wheat (*Triticum aestivum*, 19%), were the main crop during the years 2003–2005, followed by sugar beet (*Beta vulgaris*, 3%), maize (*Zea mays*, 2%), sunflower (*Helianthus annuus*, 2%), turf (2%), onion (*Allium cepa*, 1%), potato (*Solanum tuberosum*, 1%), and pea (*Pisum sativum*, 1%). Non-farmed habitat types included fallow land (10%), hedges/thickets (2%), and woodland (1%). Furthermore, over the study period, fields were covered by intertillage (14%, see Table 1 for the specific plant species), germinating seeds (4%), or were free from vegetation (bare ground, 24%; and agricultural infrastructure such as farm tracks, 5%). Hare density at the study site, estimated yearly by spring spotlight counts [27], was on average 91 individuals per 100 ha (range: 42–150).

**Table 1:** Plant taxa included in the five plant groups: cereals, intertillage, other field crops, trees/shrubs, weeds/grasses.

Weeds/grasses	Cereals	Intertillage	Other field crops	Trees/shrubs
*Amaranthus* sp.	*Hordeum vulgare*	Fagopyrum esculentum*	*Beta vulgaris*	*Cornus sanguinea*
*Anagallis arvensis*	*Secale cereale*	*Lathyrus* sp.	*Daucus carota*	*Robinia pseudoacacia*
*Arrhenatherum elatius*	*Triticum aestivum*	*Phacelia*	*Glycine max*	*Malus domestica*
*Artemisia* sp.	*Zea mays*	Pisum sativum*	*Helianthus annuus*	
*Atriplex* sp.		*Sinapis arvensis*	Pisum sativum*	
*Avena fatua*				
*Avenula pubescens*				
*Bromus* sp.				
*Capsella bursa-pastoris*				
*Carduus* sp.				
*Cichorium intybus*				
*Convolvulus arvensis*				
*Dactylis glomerata*				
*Deschampsia flexuosa*				
*Elymus repens*				
Fagopyrum esculentum*				
*Fallopia convolvulus*				
*Festuca rubra* agg				
Unidentified grass				
*Hieracium* sp.				
*Hordeum murinum*				
*Hypochaeris*				
*Juncus* sp.				
*Lactuca virosa*				
*Lamium* sp.				
*Leontodon* sp.				
*Lolium* sp.				
*Lotus* sp.				
*Medicago sativa*				
*Nardus stricta*				
*Panicum miliaceum*				
*Papaver rhoeas*				
*Plantago lanceolata*				
*Poa* sp.				
*Polygonum aviculare*				
*Rubus caesius*				
*Silene* sp.				
*Stellaria media*				
*Thymus pulegioides*				
*Trifolium incarnatum/resupinatum*				
*Trifolium pratense*				
*Trifolium repens*				
*Trifolium* sp.				
*Verbascum* sp.				
Unidentified weed				

Asterisks indicate plant species occurring in the plant groups weeds/grasses or other field crops and intertillage.

### Data collection

During the three years of the study, 570 European hares were shot during the day in February (“winter”, n = 72), May (“spring”, n = 127), August (“summer”, n = 97), and November (“autumn”, n = 274). All animals were sexed according to secondary sexual characteristics. Age was determined by the weight of the dried eye lenses [28]. Hares younger than one year of age (eye lens weight ≤ 276 mg) were classified as subadults. Stomach contents were analysed chemically and botanically (for methods see [7] (DM composition), [5] (FA composition), and [22] (botany)). We calculated gross energy (kJ/g) using energetic values given by Valencak and Ruf [7]. Stomach contents represented the food items taken up by the European hares during the previous nocturnal activity period [3]. Sample sizes vary because not all stomach contents could be analysed for both botanical and composition of nutrients.

During each season a semi-quantitative botanical inventory of the complete study area was conducted (for methods see [22]). Furthermore, a plant sample for each crop type and for fallow land was collected once per season, for which the DM and FA composition were determined (for analyses of DM composition see [7], for FA composition [5]). We assumed that the circular plot of 10 ha around the location where each hare was shot was that hare’s potential feeding area during the last 12 hours, as GPS collared hares in this study site had 24-hour home ranges of up to 10 ha [29]. Only European hares with at least 75% of their circular home ranges falling within the study area were considered representative and used for further analyses (i.e. hares shot close to the boundaries of the study area were excluded, n = 171). Thus, the botanical inventory within each circular plot represented the available forage for a hare. Estimates of the average nutrient and energy content of available forage in each plot were calculated on the basis of this inventory.

### Data analysis

Dietary preferences were measured by using Chesson’s Electivity Index ε [30], an index based on Manly’s alpha [31], which can be used to analyse dietary preferences [32]. We chose Chesson’s Electivity Index because it has the advantage that individual dietary preferences are comparable for a varying number of food types available to different individuals. The Chesson’s Electivity Index ranges between -1 and +1; negative values signify negative selection (avoidance), whereas positive values signify positive selection (preference). We calculated Chesson’s Electivity Indices for each (1) DM component (crude ash, carbohydrates, crude fat, crude fibre, and crude protein), (2) FA component (myristic acid (C14:0), palmitic acid (C16:0), palmitoleic acid (C16:1), stearic acid (C18:0), oleic acid (C18:1), LA, and ALA), (3) plant taxon, and (4) plant group (cereals, intertillage, other field crops, trees/shrubs, weeds/grasses, Table 1).

The reliability of each electivity index was tested by bootstrapping [33]. The original ε_i_ values (ε_i_ = Chesson’s Electivity Index for the food type i) were resampled 1000 times with replacement and an accelerated bootstrap confidence interval was calculated. The accelerated bootstrap adjusted the confidence interval for bias and skewness [34]. If the lower and upper 95% CI featured the same algebraic sign, the selection for this food type was significant. We only bootstrapped ε_i_ values for forage components if they were selected by 7 or more hares, as smaller sample sizes provide unreliable results. For this reason, it was not possible to bootstrap the electivity indices for seasonal differences between age classes or sexes.

The specific FA proportions are typically autocorrelated and, hence, not independent. This does not, however, prohibit statistically analysing preferences for each FA separately, it merely follows that resulting electivity indices can be expected to be similar for FA whose proportions are positively correlated.

### Statistical analyses

All analyses were computed with the software R 3.0.2 [35]. We analysed the gross energy data by linear mixed-effects modelling using the package lme4 [36]. *P*-values and parameter estimates (β) were extracted by Markov chain Monte Carlo sampling based on 10000 simulation runs [37] using restricted maximum likelihood. We visually checked the normality of the model residuals by means of a normal probability plot. Homogeneity of variances and goodness-of-fit were examined by plotting residuals versus fitted values [38]. The model included hare identity as a random factor in order to allow paired testing for the available gross energy (in the plants in the circular plot) and used gross energy (in the diet), and a specific code for the month and year as a second random factor in order to account for the different seasons and years of the study. We then tested whether there was a difference between available and used gross energy.

DM and FA components (response variables) were also analysed using linear mixed-effects models with the package lme4 [36]. The full model for the DM response variables included the covariates season (4 levels), sex, age (adult vs. subadult) and their two-way interaction terms. Moreover, the models included year as a random factor in order to account for the different years of the study. The full models were used to create a set of models with all combinations of the independent variables using the package MuMln [39]. *P*-values and estimates (β) were extracted by model averaging (including all models with delta AIC<10). The residuals of the full models were checked for normal distribution by viewing QQ-plots and histograms. Post-hoc tests with the best model were computed for the covariate season using the Tukey’s all-pair comparisons method in the package multcomp [40].

### Ethics Statement

Hares were shot by local hunters using rifles in winter, spring and summer. In autumn, hares were collected on regular hunts using shotguns. Outside the legal hunting time (autumn), local hunters were given permission to shoot hares by the local administration of Gänserndorf, Lower Austria. The method of collecting hares was not specified, but shooting was conducted according to hunting law (using rifles on single hunts and shotguns on drive hunts). Collecting hares outside the legal hunting period was permitted on the basis of the Lower Austrian Hunting Law §§ 75 and 76, which allow exceptions to regular hunting periods for scientific research. Notification by the local administration of Gänserndorf was given on the 20th of January 2003. The land accessed was not protected and no protected species were sampled. The study complies with the current laws of Austria.

## Results

Chemical preferences for DM and gross energy were assessed in 263 hares (122 males, 141 females), whereas the FA preferences of 269 hares (124 males, 145 females) were evaluated. The stomach contents of 382 hares (141 males, 241 females) were analysed botanically.

### Selection for energy and DM

**Fig 1:**
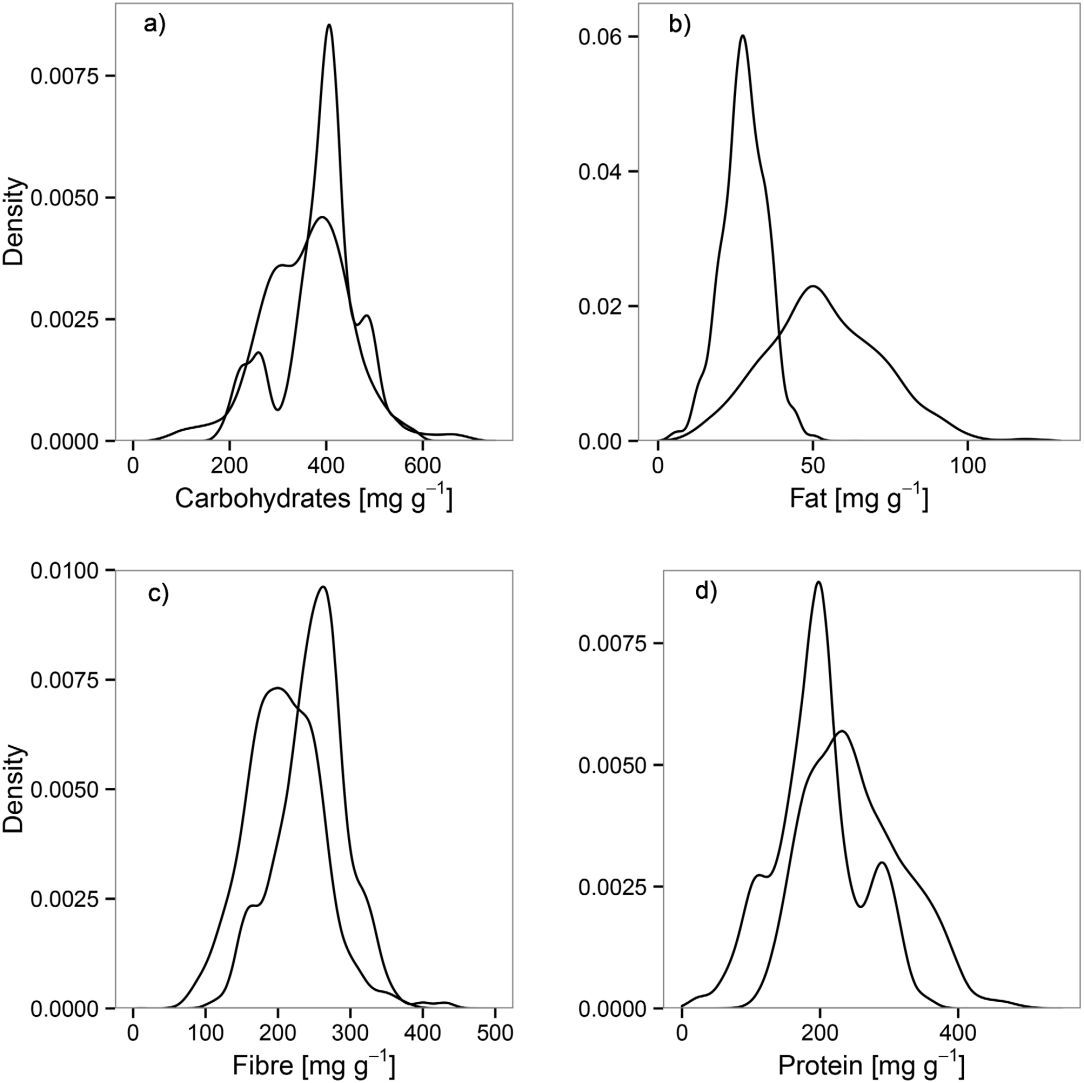
Kernel density estimates for dry matter components available and used by European hares. Kernel density estimates for dry matter (DM) components (a) carbohydrates, (b) crude fat, (c) crude fibre and (d) crude protein [mg/g], available to and used by European hares (n = 263). Used components (in the diet) are indicated in white; available components (in the forage sampled in the habitat) are indicated by grey shading.

We found a significant difference between the gross energy of available (mean 12.450 kJ/g; SE ± 0.057) and used (mean 14.356 kJ/g; SE ± 0.072) forage plants (*p*_*MCMC*_ = 0.0001; β = 0.126), showing that our study animals selected their food for high energy content. Hares preferred plants rich in crude fat and crude protein; all other DM components were avoided throughout the year (see S1 Table). The animals mostly avoided crude fibre in their diet, whereas crude fat was preferred the most. Our results show a distinct difference between the availability of crude fat in the environment and its use in the diet of European hares (Fig 1b). The variance between available and used crude protein and crude fibre was less pronounced, and it was almost non-existent for carbohydrates (Fig 1a, 1c and 1d). Furthermore, we found significant differences in the selection of all DM components between the seasons (Table 2). Although seasonal differences were recorded, the general preference for crude fat and the general avoidance of carbohydrates were maintained throughout the year (see S1 Table). However, crude protein was significantly preferred in autumn and winter, while it was not significantly selected in spring and summer. We found no significant effects of sex (*p* > 0.05) or age class (*p* > 0.05) on any DM component (see S2 Table).

**Table 2:** Post-hoc test results of the electivity indices (parameter estimates β and *p*-values) of all DM components for the covariate season using the Tukey’s all-pair comparisons method (n = 263).

	Ash		Carbohydrates		Fat		Fibre		Protein	
	β	*p*	β	*p*	β	*p*	Β	*p*	β	*p*
Winter-Summer	0.322	<0.001	-0.174	0.001	-0.231	<0.001	-0.031	0.923	0.099	0.037
Spring-Summer	0.208	<0.001	0.045	0.683	-0.082	0.013	-0.013	0.990	-0.005	0.998
Autumn-Summer	0.309	<0.001	-0.111	0.076	-0.106	0.001	-0.142	0.015	0.100	0.007
Spring-Winter	-0.114	0.066	0.219	<0.001	0.149	<0.001	0.018	0.977	-0.104	0.017
Autumn-Winter	-0.012	0.996	0.062	0.599	0.125	0.001	-0.111	0.110	0.001	1.000
Autumn-Spring	0.101	0.159	-0.157	0.002	-0.024	0.815	-0.129	0.013	0.105	0.002

### Selection for certain FA

**Fig 2:**
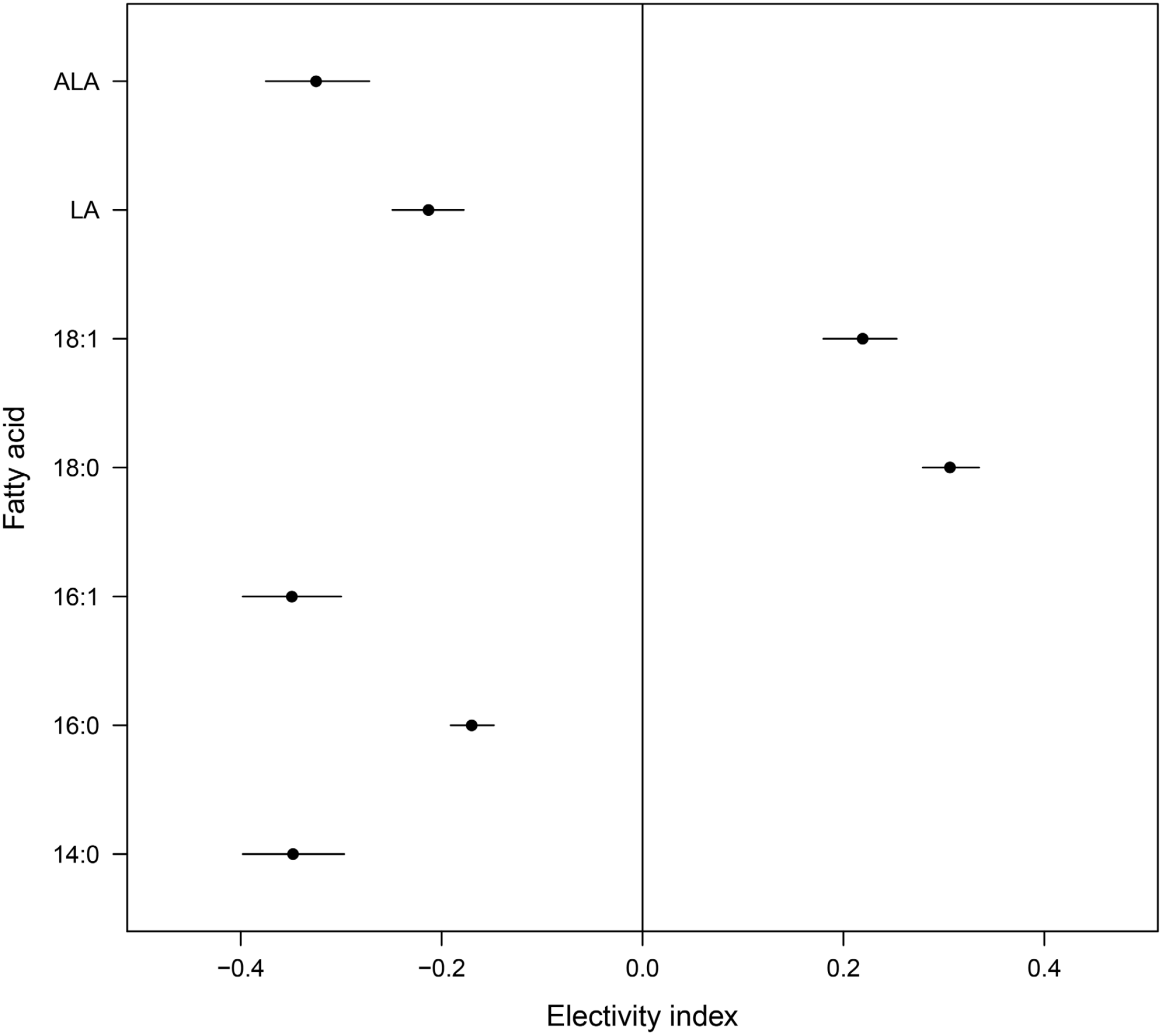
Chesson’s Electivity Indices of the different fatty acids. Chesson’s Electivity Indices in European hares (n = 269) and their distributions of 1000 bootstrap resamples (mean and 95% confidence interval) of the different fatty acids (FA). See text for details of statistics.

Hares avoided the FA 14:0, 16:0, 16:1, LA and ALA, whereas 18:0 and 18:1 were preferred throughout the year (Fig 2). Selection for every FA was influenced by the season (Table 3), but general avoidance or preference was maintained in the course of the year (see S1 Table). Furthermore, sex and age had a significant influence on ALA selection (sex: *p* = 0.016; β_male_ = -0.138, age: *p* = 0.077; β_subadult_ = 0.102, Table 4), i.e. females and subadults had higher ALA intake. Intake of none of the other FA was significantly influenced by sex (*p* > 0.10) or age (*p* > 0.10, see Table 5 for LA).

**Table 3:** Post-hoc test results of the electivity indices (parameter estimates β and *p*-values) of the different fatty acids for the covariate season using the Tukey’s all-pair comparisons method (n = 269).

	FA 14:0		FA 16:0		FA 16:1		FA 18:0		FA 18:1		LA		ALA	
	β	*p*	Β	*p*	β	*p*	β	*p*	β	*p*	β	*p*	β	*p*
Winter-Summer	0.647	<0.001	0.059	0.271	-0.109	0.352	0.155	0.117	0.121	0.151	-0.356	<0.001	-0.527	<0.001
Spring-Summer	0.113	0.164	-0.062	0.095	0.137	0.043	-0.032	0.941	0.136	0.014	-0.050	0.694	-0.080	0.413
Autumn-Summer	0.664	<0.001	-0.198	<0.001	0.170	0.013	0.198	<0.001	0.192	<0.001	-0.567	<0.001	-0.716	<0.001
Spring-Winter	-0.534	<0.001	-0.121	<0.001	0.246	<0.001	-0.187	0.052	0.015	0.992	0.305	<0.001	0.447	<0.001
Autumn-Winter	0.016	0.994	-0.257	<0.001	0.278	<0.001	0.043	0.916	0.071	0.592	-0.211	<0.001	-0.190	0.029
Autumn-Spring	0.551	<0.001	-0.136	<0.001	0.033	0.937	0.230	<0.001	0.056	0.585	-0.516	<0.001	-0.636	<0.001

**Table 4:** Model averaged coefficients for the response variable ALA (n = 269).

	Estimate	Std. Error	z value	*p*
Intercept	0.029	0.091	0.321	0.749
Subadult	0.102	0.058	1.767	0.077
Winter	-0.549	0.093	5.924	<0.001
Spring	-0.073	0.062	1.174	0.240
Autumn	-0.711	0.064	11.054	<0.001
Male	-0.138	0.057	2.404	0.016
Subadult:Male	0.061	0.086	0.714	0.475
Winter:Male	0.203	0.127	1.603	0.109
Spring:Male	-0.033	0.101	0.322	0.747
Autumn:Male	0.086	0.151	0.568	0.570
Subadult: Winter	-0.176	0.168	1.045	0.296
Subadult:Spring	-0.003	0.105	0.028	0.978
Subadult:Autumn	-0.143	0.106	1.354	0.176

The intercept stands for the estimate for female adults in summer.

**Table 5:** Model averaged coefficients for the response variable LA (n = 269).

	Estimate	Std. Error	z value	*p*
Intercept	-0.013	0.035	0.369	0.712
Subadult	0.052	0.031	1.649	0.100
Winter	-0.350	0.059	5.881	<0.001
Spring	-0.059	0.053	1.116	0.264
Autumn	-0.569	0.039	14.695	<0.001
Male	0.078	0.047	1.650	0.100
Winter:Male	0.070	0.077	0.908	0.364
Spring:Male	-0.162	0.062	2.619	0.009
Autumn:Male	0.035	0.092	0.386	0.699
Subadult:Male	-0.058	0.052	1.115	0.265
Subadult: Winter	0.040	0.105	0.380	0.704
Subadult:Spring	-0.036	0.064	0.557	0.577
Subadult:Autumn	0.025	0.066	0.374	0.709

The intercept stands for the estimate for female adults in summer.

### Selection for plant taxa

**Fig 3:**
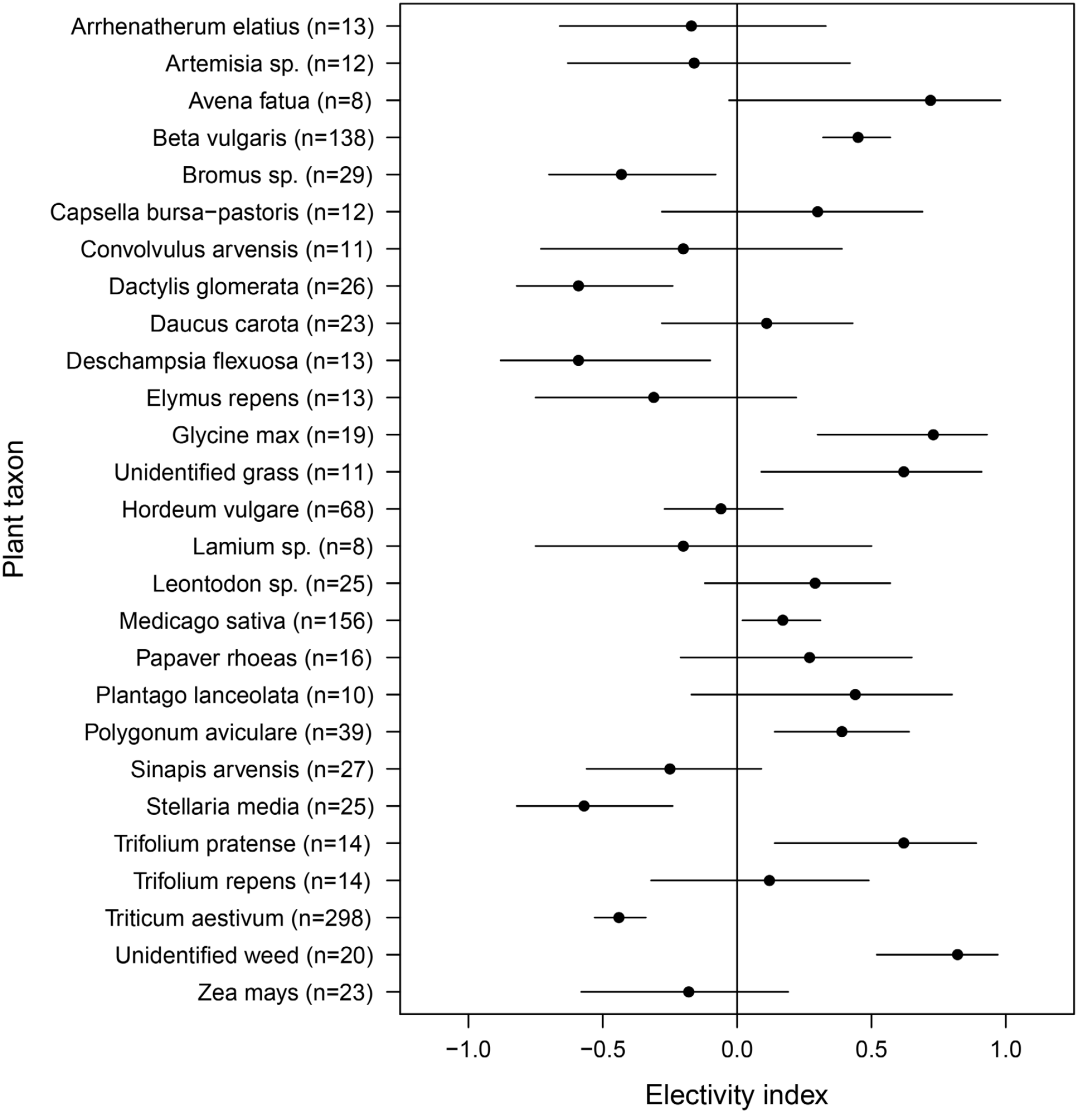
Chesson’s Electivity Indices for plant taxa. Chesson’s Electivity Indices in European hares (n = 399) and their distributions of 1000 bootstrap resamples (mean and 95% confidence interval) for plant taxa selected by n≥7 hares (sample size in brackets is the number of hares selecting each plant taxon). Non-significant results cross the vertical line at zero. See text for details of statistics.

Hares selected several plant taxa, but the pattern of selectivity changed over the course of the year (Fig 3). Favoured plant taxa in winter were sugar beet roots (*Beta vulgaris*, ɛ = 0.48, n = 25) and lucerne (*Medicago sativa*, ɛ = 0.55, n = 20), whereas in spring soybean (*Glycine max*, ɛ = 0.82, n = 12) was preferred. In summer, the European hares did not show any preferences for any plant taxon. In autumn, the hares chose sugar beet (ɛ = 0.77, n = 57), unidentified weed (ɛ = 0.91, n = 9), barley (*Hordeum vulgare*, ɛ = 0.83, n = 7), lucerne (ɛ = 0.24, n = 67), chickweed (*Stellaria media*, ɛ = 0.60, n = 8), and maize (*Zea mays*, ɛ = 0.67, n = 7).

**Fig 4:**
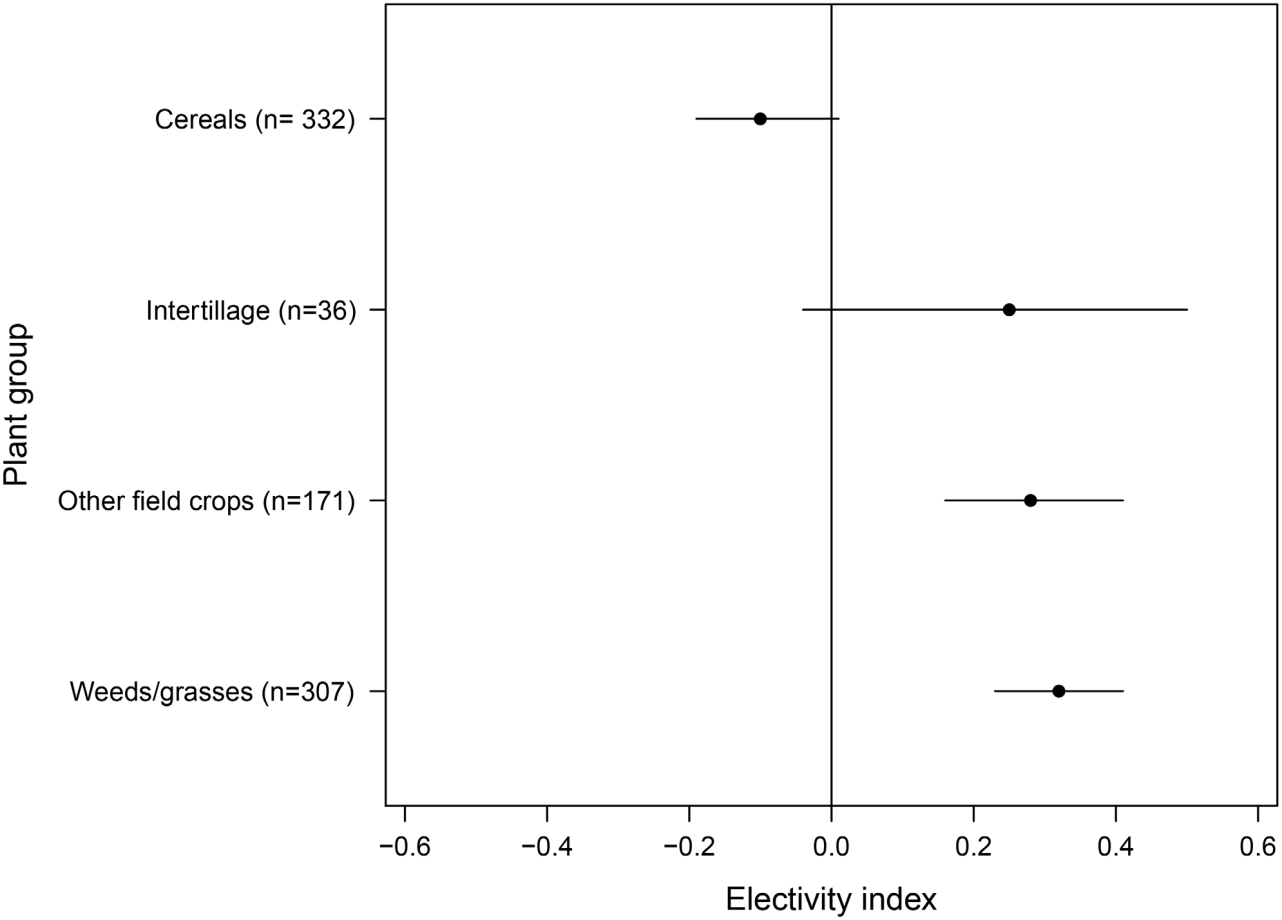
Chesson’s Electivity Indices for plant groups. Chesson’s Electivity Indices in European hares (n = 399) and their distributions of 1000 bootstrap resamples (mean and 95% confidence interval) for plant groups which were selected by n≥7 hares (sample size in brackets is the number of hares selecting the respective plant groups). Non-significant results cross the vertical line at zero. See text for details of statistics.

When grouping plant taxa to cereals, intertillage, other field crops, and weeds/grasses we determined a preference for weeds/grasses and other field crops (Fig 4). In the course of the year, other field crops were preferred in winter (ɛ = 0.87, n = 32), weeds/grasses in spring (ɛ = 0.64, n = 72) and summer (ɛ = 0.34, n = 68), and both plant groups were positively selected in autumn (other field crops: ɛ = 0.62, n = 58; weeds/grasses: ɛ = 0.26, n = 133).

## Discussion

In previous studies, we showed that the reproductive performance of captive female European hares [2] and leveret growth and survival [1] are enhanced when the females are fed a diet rich in energy. The results of the present study are consistent with previous studies, and support our hypothesis (1): that European hares in the wild choose their diet for high energy intake. Hares preferred a diet rich in crude fat, irrespective of its content in specific FA. Hares did not show random dietary uptake. Rather, they are highly selective feeders, so we would not expect random sampling of plant nutrient composition in an area to reflect adequately the diet of foraging wild hares. This selectivity may explain why previous researchers [17–18] were unable to show an effect of mean food quality in hare habitats on life history traits and density in European hares. As well as female and subadult hares, males also preferred a high energy diet. Although the energy costs of lactation in females and growth of young are comparably high, we demonstrate that all hares, irrespective of age and sex, prefer plants rich in crude fat. Selecting an energy-rich diet may reduce gut content weight, thus enabling hares to run faster and escape from predators while meeting their daily energy demands. Moreover, in our continental study site in Lower Austria, where precipitation is less than 500 mm per year (see also [41]), a shortage of water in the diet might be compensated for by oxygenizing fat and producing metabolic water [42].

Our hypothesis (3), that dietary preferences vary according to season, individual age and sex, can be rejected with respect to age and sex effects. However, we did find seasonal effects: the preference for a diet rich in crude fat was highest in summer, when the hares’ mating season in Central Europe is gradually coming to an end [43]. Furthermore, our data indicate that hares build up their fat reserves in winter: when fat stores were fully built up in winter, our European hares showed the least selection for crude fat in their diet (ε crude fat: winter = 0.13, spring = 0.28, summer = 0.36, autumn = 0.26, see S1 Table). This is in accordance with Zörner, Flux and Popescu et al. [3–5].

We did not find support for our hypothesis (2), that European hares preferred plants particularly rich in LA and ALA. Both FA were not preferred across seasons and study years. This may not indicate an active avoidance, but may result instead from preferences for other plant properties.

The high PUFA content in hares’ body tissues [15] seems to be solely obtained by their targeted utilisation of the available PUFA in the gastrointestinal tract [5], and not, as suggested in the current literature, by selective uptake through the diet (see [44] for a review). Consequently, we suggest that the European hare’s diet selection is adapted to maximise energy intake by taking a diet rich in fat. Simultaneously, requirements of specific FA are covered by physiological mechanisms in the gastrointestinal tract that allow the selective absorption of PUFA. Only during lactation, when the supply of certain PUFA such as LA is crucial [9], European hares positively select plants rich in LA as indicated by an increased content of LA in the gastrointestinal tract contents of lactating females [5].

Our results support the view that European hares prefer certain plant taxa. Of the 349 different plant taxa identified in the study area, 47 were found in the hares’ stomachs, and only 10 of those were positively selected for by the European hares throughout the year or in the whole study period (*Beta vulgaris*, *Glycine max*, *Hordeum vulgare*, *Medicago sativa*, *Polygonum aviculare*, *Stellaria media*, *Trifolium pratense*, *Zea mays*, unidentified weed, and unidentified grass; see S1 Table). The preferred plant taxa in all our study years (2003–2005) were only partly in line with previous findings for 2003 only at the same study site [22]. *Beta vulgaris* roots in winter, *Glycine max* in spring and *Beta vulgaris* roots in autumn were preferred consistently, regardless of whether the analysis was conducted for the year 2003 only or for the whole study period. However, whereas *Triticum aestivum* was positively selected in autumn in 2003 [22], the entire data set (2003–2005) revealed that the hares avoided this field crop during all the four seasons. We believe that these inconsistencies are either due to the comparably small sample size for 2003 which did not allow rigorous statistical analysis [22] (2003: n = 117; 2003–2005: n = 382), or due to the different circular plot sizes used to represent the available food items for each hare. We used small potential feeding plots of 10 ha for the analysis of the 2003–2005 data, but Reichlin et al. calculated electivity indices on the basis of much larger potential feeding sites (50 ha) for the 2003 data [22]. When plant taxa were pooled into groups for analysis, we found that hares preferred weeds/grasses and other field crops over the cereals which characterize arable land in our study site (28% cover).

We expected the plant taxa selected by hares to be rich in crude fat. *Glycine max* was rich in crude fat, but *Beta vulgaris* and *Trifolium pratense* were poor in crude fat (see S3 Table). Similarly inconsistent results were recorded for the plant groups: the category other field crops was high in crude fat but weeds/grasses was low in crude fat. If hares select for crude fat, but the plant taxa they select are not *per se* rich in crude fat, then this suggests that hares are selecting high-fat plant parts which greatly exceed the average crude fat value of the plant. This is in concordance with the finding that hares select specific plants, although the average energy content of the plants in a habitat might be low (see above).

The selective feeding of European hares on energy sources which include non-crop plants supports the call to increase habitat heterogeneity, crop diversity and unused fields such as set-asides and fallow land [45, 2, 46–47]. Our findings suggest that, in order to increase the supply of preferred forage plants for the European hare in arable land, landscape structures such as field margins, fallow land, and buffer strips containing significant fractions of weeds and grasses should be created in preference to hedges, trees and afforested areas. To enhance arable biodiversity and provide a variety of the European hare’s preferred food plants, different types of non-farmed features should be promoted. Suitable target areas for habitat improvement are: (1) areas of reduced arable productivity because of shadow effects, such as strips adjacent to woods or shelter belts, (2) areas that are already out of production, such as strips adjacent to water, and (3) strips between conventionally and organically farmed fields. Optimal set-asides for European hares should include weeds such as *Medicago sativa*, *Polygonum aviculare*, *Stellaria media*, *Glycine max* and *Trifolium pratense* as well as grasses. Moreover, the vegetation of the non-farmed features should be kept low and sparse to enhance its suitability for foraging hares [48]. Our results show that European hares are highly selective in their food choice during winter. Improved food availability for European hares in this season might be achieved by encouraging farmers to sow set-aside and field margin strips in early autumn, which will provide fresh fodder in late autumn and early winter. The proportion of land planted with wildlife-friendly seed mixtures or set-asides needs to be high (at least 14%), in order to be beneficial [49], and in order to avoid concentration of hares in a few areas, as this leads to intra-specific stress [50] and higher disease transmission (e.g. [51]). In addition, predators may search these areas systematically, especially if they are arranged as linear structures, which may reverse the positive effects of habitat improvements [52]. In the light of recent declines in populations of European hares throughout Europe [45], these recommendations for agri-environment schemes are important steps towards the evidence-based conservation of the species in intensively farmed arable land.

## Supporting Information

S1 Table Electivity Indices for each DM and FA component, plant taxon and plant group. (DOC)

S2 Table Model averaged coefficients for the response variables ash, carbohydrates, crude fat, crude fibre, crude protein, FA 14:0, FA 16:0, FA 16:1, FA 18:0 and FA 18:1. (DOC)

S3 Table Mean DM and FA content for each plant taxon and plant group. (DOC)
