# Supplementary material for: The European Hare (Lepus europaeus): A Picky Herbivore Searching for Plant Parts Rich in Fat
Source: PLoS One. 2015 Jul 31;10(7):e0134278. doi: 10.1371/journal.pone.0134278 (PMC4521881; doi:10.1371/journal.pone.0134278)
Supplement: S1 Table — (DOC) [file pone.0134278.s001.doc]

**S1**

Chesson’s Electivity Indices and sample size for each DM and FA component, plant taxon and plant group pooled over the three study years 2003-2005 and for each season separately (winter, spring, summer, autumn). In addition, Chesson’s Electivity Indices are indicated as separate calculations for different sex and age groups (subadult, adult) of the animals. Confinements are marked with the abbreviation n.s. to indicate non-significant results and with n≤7 for not reliable bootstrap results.

| Timeframe | Dry matter | ɛ | n | Confinement |
| --- | --- | --- | --- | --- |
| Whole study period | Ash | -0.20 | 262 |  |
|  | Carbohydrates | -0.16 | 262 |  |
|  | Crude fat | 0.27 | 263 |  |
|  | Crude fibre | -0.21 | 263 |  |
|  | Crude protein | 0.05 | 263 |  |
| Winter | Ash | -0.04 | 40 | n.s. |
|  | Carbohydrates | -0.26 | 40 |  |
|  | Crude fat | 0.13 | 40 |  |
|  | Crude fibre | -0.23 | 40 |  |
|  | Crude protein | 0.13 | 40 |  |
| Spring | Ash | -0.18 | 85 |  |
|  | Carbohydrates | -0.08 | 85 |  |
|  | Crude fat | 0.28 | 85 |  |
|  | Crude fibre | -0.19 | 85 |  |
|  | Crude protein | 0.01 | 85 | n.s. |
| Summer | Ash | -0.34 | 70 |  |
|  | Carbohydrates | -0.18 | 70 |  |
|  | Crude fat | 0.36 | 70 |  |
|  | Crude fibre | -0.19 | 70 |  |
|  | Crude protein | 0.01 | 70 | n.s. |
| Autumn | Ash | -0.18 | 67 |  |
|  | Carbohydrates | -0.18 | 67 |  |
|  | Crude fat | 0.26 | 68 |  |
|  | Crude fibre | -0.25 | 68 |  |
|  | Crude protein | 0.11 | 68 |  |

| Timeframe | Fatty acid | ɛ | n | Confinement |
| --- | --- | --- | --- | --- |
| Whole study period | FA 14:0 | -0.35 | 269 |  |
|  | FA 16:0 | -0.17 | 269 |  |
|  | FA 16:1 | -0.35 | 269 |  |
|  | FA 18:0 | 0.31 | 269 |  |
|  | FA 18:1 | 0.22 | 269 |  |
|  | LA | -0.21 | 269 |  |
|  | ALA | -0.33 | 269 |  |
| Winter | FA 14:0 | 0.07 | 43 | n.s. |
|  | FA 16:0 | -0.07 | 43 | n.s. |
|  | FA 16:1 | -0.68 | 43 |  |
|  | FA 18:0 | 0.32 | 43 |  |
|  | FA 18:1 | 0.21 | 43 |  |
|  | LA | -0.26 | 43 |  |
|  | ALA | -0.53 | 43 |  |
| Spring | FA 14:0 | -0.51 | 85 |  |
|  | FA 16:0 | -0.18 | 85 |  |
|  | FA 16:1 | -0.26 | 85 |  |
|  | FA 18:0 | 0.28 | 85 |  |
|  | FA 18:1 | 0.22 | 85 |  |
|  | LA | -0.12 | 85 |  |
|  | ALA | -0.14 | 85 |  |
| Summer | FA 14:0 | -0.65 | 73 |  |
|  | FA 16:0 | -0.11 | 73 |  |
|  | FA 16:1 | -0.38 | 73 |  |
|  | FA 18:0 | 0.21 | 73 |  |
|  | FA 18:1 | 0.17 | 73 |  |
|  | LA | 0.03 | 73 | n.s. |
|  | ALA | -0.07 | 73 | n.s. |
| Autumn | FA 14:0 | -0.09 | 68 |  |
|  | FA 16:0 | -0.29 | 68 |  |
|  | FA 16:1 | -0.23 | 68 |  |
|  | FA 18:0 | 0.43 | 68 |  |
|  | FA 18:1 | 0.28 | 68 |  |
|  | LA | -0.56 | 68 |  |
|  | ALA | -0.70 | 68 |  |

| Timeframe | Plant taxon | ɛ | n | Confinement |
| --- | --- | --- | --- | --- |
| Whole study period | *Amaranthus sp.* | -0.03 | 6 | n.s. |
|  | *Arrhenatherum elatius* | -0.17 | 13 | n.s. |
|  | *Artemisia sp.* | -0.16 | 12 | n.s. |
|  | *Atriplex sp.* | -0.14 | 2 | n≤7 |
|  | *Avena fatua* | 0.72 | 8 | n.s. |
|  | *Beta vulgaris* | 0.45 | 138 |  |
|  | *Bromus sp.* | -0.43 | 29 |  |
|  | *Capsella bursa-pastoris* | 0.30 | 12 | n.s. |
|  | *Cichorium intybus* | 0.94 | 3 | n≤7 |
|  | *Convolvulus arvensis* | -0.20 | 11 | n.s. |
|  | *Dactylis glomerata* | -0.59 | 26 |  |
|  | *Daucus carota* | 0.11 | 23 | n.s. |
|  | *Deschampsia flexuosa* | -0.59 | 13 |  |
|  | *Elymus repens* | -0.31 | 13 | n.s. |
|  | *Fagopyrum esculentum* | 0.98 | 4 | n≤7 |
|  | *Fallopia convolvulus* | 0.03 | 4 | n.s. |
|  | *Festuca rubra agg* | -0.02 | 5 | n.s. |
|  | *Glycine max* | 0.73 | 19 |  |
|  | Unidentified grass | 0.62 | 11 |  |
|  | *Helianthus annuus* | 1.00 | 2 | n≤7 |
|  | *Hieracium* | 0.91 | 3 | n≤7 |
|  | *Hordeum vulgare* | -0.06 | 68 | n.s. |
|  | *Juncus sp.* | -0.51 | 2 | n≤7 |
|  | *Lamium* | -0.20 | 8 | n.s. |
|  | *Lathyrus* | -0.94 | 5 | n≤7 |
|  | *Leontodon sp.* | 0.29 | 25 | n.s. |
|  | *Lotus sp.* | -0.50 | 4 | n.s. |
|  | *Medicago sativa* | 0.17 | 156 |  |
|  | *Nardus stricta* | 0.97 | 2 | n≤7 |
|  | *Panicum miliaceum* | 0.98 | 3 | n≤7 |
|  | *Papaver rhoeas* | 0.27 | 16 | n.s. |
|  | *Phacelia* | -0.77 | 5 | n≤7 |
|  | *Plantago lanceolata* | 0.44 | 10 | n.s. |
|  | *Poa sp.* | 0.30 | 5 | n.s. |
|  | *Polygonum aviculare* | 0.39 | 39 |  |
|  | *Robinia pseudoacacia* | 0.96 | 3 | n≤7 |
|  | *Rubus caesius* | -0.44 | 3 | n.s. |
|  | *Secale cereale* | -0.01 | 4 | n.s. |
|  | *Sinapis arvensis* | -0.25 | 27 | n.s. |
|  | *Stellaria media* | -0.57 | 25 |  |
|  | *Trifolium incarnatum/resupinatum* | 0.48 | 3 | n.s. |
|  | *Trifolium pratense* | 0.62 | 14 |  |
|  | *Trifolium repens* | 0.12 | 14 | n.s. |
|  | *Triticum aestivum* | -0.44 | 298 |  |
|  | *Verbascum sp.* | 0.98 | 2 | n≤7 |
|  | Unidentified weed | 0.82 | 20 |  |
|  | *Zea mays* | -0.18 | 23 | n.s. |
| Winter | *Beta vulgaris* | 0.48 | 25 |  |
|  | *Bromus sp.* | -0.09 | 2 | n≤7 |
|  | *Capsella bursa-pastoris* | 0.54 | 6 | n.s. |
|  | *Dactylis glomerata* | -0.07 | 2 | n.s. |
|  | *Daucus carota* | 0.27 | 15 | n.s. |
|  | Unidentified grass | -0.07 | 2 | n.s. |
|  | *Juncus sp.* | -0.46 | 2 | n≤7 |
|  | *Lamium sp.* | -0.60 | 3 | n≤7 |
|  | *Medicago sativa* | 0.55 | 20 |  |
|  | *Polygonum aviculare* | -0.44 | 2 | n≤7 |
|  | *Sinapis arvensis* | 0.23 | 9 | n.s. |
|  | *Stellaria media* | 0.00 | 13 | n.s. |
|  | *Triticum aestivum* | -0.88 | 38 |  |
|  | *Zea mays* | 0.04 | 5 | n.s. |
| Spring | *Arrhenatherum elatius* | -0.19 | 5 | n.s. |
|  | *Avena fatua* | 1.00 | 2 | n≤7 |
|  | *Beta vulgaris* | -0.21 | 18 | n.s. |
|  | *Bromus sp.* | -0.44 | 12 |  |
|  | *Capsella bursa-pastoris* | -0.29 | 2 | n.s. |
|  | *Convolvulus arvensis* | 0.50 | 3 | n.s. |
|  | *Dactylis glomerata* | 0.07 | 9 | n.s. |
|  | *Elymus repens* | 0.60 | 5 | n.s. |
|  | *Glycine max* | 0.82 | 12 |  |
|  | Unidentified grass | 0.95 | 3 | n≤7 |
|  | *Helianthus annuus* | 1.00 | 2 | n≤7 |
|  | *Hordeum vulgare* | -0.36 | 29 | n.s. |
|  | *Lamium sp.* | 1.00 | 2 | n≤7 |
|  | *Leontodon sp.* | 0.35 | 7 | n.s. |
|  | *Medicago sativa* | 0.26 | 28 | n.s. |
|  | *Papaver rhoeas* | 0.32 | 13 | n.s. |
|  | *Plantago lanceolata* | 0.59 | 3 | n≤7 |
|  | *Polygonum aviculare* | 0.40 | 4 | n.s. |
|  | *Rubus caesius* | -0.42 | 3 | n.s. |
|  | *Secale cereale* | -0.03 | 4 | n.s. |
|  | *Stellaria media* | -0.05 | 5 | n.s. |
|  | *Trifolium pratense* | 0.41 | 11 | n.s. |
|  | *Trifolium repens* | 0.23 | 8 | n.s. |
|  | *Triticum aestivum* | -0.55 | 68 |  |
|  | Unidentified weed | 0.76 | 6 | n≤7 |
|  | *Zea mays* | -0.06 | 3 | n.s. |
| Summer | *Amaranthus sp.* | 0.61 | 3 | n≤7 |
|  | *Arrhenatherum elatius* | 0.20 | 4 | n.s. |
|  | *Artemisia sp.* | 0.23 | 9 | n.s. |
|  | *Atriplex sp.* | -0.09 | 2 | n≤7 |
|  | *Beta vulgaris* | -0.30 | 40 |  |
|  | *Bromus sp.* | 0.90 | 2 | n≤7 |
|  | *Convolvulus arvensis* | 0.60 | 4 | n≤7 |
|  | *Dactylis glomerata* | -0.08 | 11 | n.s. |
|  | *Daucus carota* | 0.24 | 6 | n.s. |
|  | *Deschampsia flexuosa* | 0.45 | 5 | n.s. |
|  | *Elymus repens* | -0.11 | 5 | n.s. |
|  | *Fagopyrum esculentum* | 0.95 | 2 | n≤7 |
|  | *Glycine max* | 0.37 | 6 | n.s. |
|  | Unidentified grass | -0.16 | 2 | n≤7 |
|  | *Hordeum vulgare* | -0.15 | 33 | n.s. |
|  | *Lathyrus sp.* | -0.68 | 5 | n≤7 |
|  | *Leontodon sp.* | 0.14 | 8 | n.s. |
|  | *Lotus sp.* | -0.36 | 3 | n.s. |
|  | *Medicago sativa* | 0.25 | 46 | n.s. |
|  | *Panicum miliaceum* | 0.97 | 2 | n≤7 |
|  | *Papaver rhoeas* | 0.85 | 2 | n≤7 |
|  | *Polygonum aviculare* | 0.43 | 5 | n.s. |
|  | *Trifolium incarnatum/resupinatum* | 0.43 | 3 | n.s. |
|  | *Triticum aestivum* | -0.31 | 46 |  |
|  | Unidentified weed | 0.79 | 5 | n≤7 |
|  | *Zea mays* | -0.56 | 8 | n.s. |
| Autumn | *Amaranthus sp.* | 0.67 | 2 | n≤7 |
|  | *Arrhenatherum elatius* | -0.23 | 5 | n.s. |
|  | *Artemisia sp.* | 0.22 | 3 | n.s. |
|  | *Avena fatua* | 0.51 | 5 | n.s. |
|  | *Beta vulgaris* | 0.77 | 57 |  |
|  | *Bromus sp.* | -0.55 | 13 |  |
|  | *Capsella bursa-pastoris* | 0.42 | 4 | n.s. |
|  | *Cichorium intybus* | 0.92 | 2 | n≤7 |
|  | *Convolvulus arvensis* | -0.05 | 4 | n.s. |
|  | *Dactylis glomerata* | 0.09 | 5 | n.s. |
|  | *Deschampsia flexuosa* | 0.07 | 7 | n.s. |
|  | *Elymus repens* | -0.09 | 3 | n.s. |
|  | *Fagopyrum esculentum* | 0.71 | 2 | n≤7 |
|  | *Fallopia convolvulus* | -0.39 | 3 | n.s. |
|  | *Festuca rubra agg* | -0.17 | 5 | n.s. |
|  | Unidentified grass | 0.89 | 5 | n≤7 |
|  | *Hieracium sp.* | 0.89 | 3 | n≤7 |
|  | *Hordeum vulgare* | 0.83 | 7 | n≤7 |
|  | *Lamium sp.* | -0.50 | 4 | n.s. |
|  | *Leontodon sp.* | -0.28 | 12 | n.s. |
|  | *Medicago sativa* | 0.24 | 67 |  |
|  | *Nardus stricta* | 0.95 | 2 | n≤7 |
|  | *Phacelia sp.* | -0.50 | 5 | n.s. |
|  | *Plantago lanceolata* | 0.78 | 5 | n≤7 |
|  | *Poa sp.* | 0.11 | 3 | n.s. |
|  | *Polygonum aviculare* | 0.31 | 29 | n.s. |
|  | *Robinia pseudoacacia* | 0.93 | 2 | n≤7 |
|  | *Sinapis arvensis* | 0.32 | 19 | n.s. |
|  | *Stellaria media* | 0.60 | 8 |  |
|  | *Trifolium pratense* | 0.95 | 3 | n≤7 |
|  | *Trifolium repens* | 0.55 | 5 | n≤7 |
|  | *Triticum aestivum* | -0.53 | 152 |  |
|  | *Verbascum* | 0.90 | 2 | n≤7 |
|  | Unidentified weed | 0.91 | 9 |  |
|  | *Zea mays* | 0.67 | 7 | n≤7 |

| Timeframe | Plant group | ɛ | n | Confinement |
| --- | --- | --- | --- | --- |
| Whole study period | Cereals | -0.10 | 332 | n.s. |
|  | Intertillage | 0.25 | 36 | n.s. |
|  | Other field crops | 0.28 | 171 |  |
|  | Trees/shrubs | -0.15 | 5 | n.s. |
|  | Weeds/grasses | 0.32 | 307 |  |
| Winter | Cereals | -0.77 | 38 |  |
|  | Intertillage | 0.22 | 9 | n.s. |
|  | Other field crops | 0.87 | 32 |  |
|  | Trees/shrubs | -0.81 | 2 | n≤7 |
|  | Weeds/grasses | -0.15 | 34 | n.s. |
| Spring | Cereals | -0.01 | 80 | n.s. |
|  | Other field crops | 0.05 | 32 | n.s. |
|  | Weeds/grasses | 0.64 | 72 |  |
| Summer | Cereals | 0.03 | 61 | n.s. |
|  | Intertillage | 0.16 | 5 | n.s. |
|  | Other field crops | -0.34 | 49 |  |
|  | Weeds/grasses | 0.34 | 68 |  |
| Autumn | Cereals | -0.03 | 153 | n.s. |
|  | Intertillage | 0.28 | 22 | n.s. |
|  | Other field crops | 0.62 | 58 |  |
|  | Trees/shrubs | -0.06 | 2 | n≤7 |
|  | Weeds/grasses | 0.26 | 133 |  |

| Timeframe | Sex | Dry matter | ɛ | n | Confinement |
| --- | --- | --- | --- | --- | --- |
| Whole study period | f | Ash | -0.23 | 140 |  |
|  | m | Ash | -0.16 | 122 |  |
|  | f | Carbohydrates | -0.18 | 140 |  |
|  | m | Carbohydrates | -0.14 | 122 |  |
|  | f | Crude fat | 0.28 | 141 |  |
|  | m | Crude fat | 0.27 | 122 |  |
|  | f | Crude fibre | -0.21 | 141 |  |
|  | m | Crude fibre | -0.22 | 122 |  |
|  | f | Crude protein | 0.08 | 141 |  |
|  | m | Crude protein | 0.03 | 122 | n.s. |
| Winter | f | Ash | -0.11 | 14 | n.s. |
|  | m | Ash | 0.00 | 26 | n.s. |
|  | f | Carbohydrates | -0.25 | 14 |  |
|  | m | Carbohydrates | -0.27 | 26 |  |
|  | f | Crude fat | 0.10 | 14 |  |
|  | m | Crude fat | 0.15 | 26 |  |
|  | f | Crude fibre | -0.22 | 14 |  |
|  | m | Crude fibre | -0.23 | 26 |  |
|  | f | Crude protein | 0.16 | 14 |  |
|  | m | Crude protein | 0.11 | 26 |  |
| Spring | f | Ash | -0.27 | 28 |  |
|  | m | Ash | -0.13 | 57 |  |
|  | f | Carbohydrates | -0.06 | 28 |  |
|  | m | Carbohydrates | -0.09 | 57 |  |
|  | f | Crude fat | 0.30 | 28 |  |
|  | m | Crude fat | 0.27 | 57 |  |
|  | f | Crude fibre | -0.15 | 28 |  |
|  | m | Crude fibre | -0.21 | 57 |  |
|  | f | Crude protein | 0.02 | 28 | n.s. |
|  | m | Crude protein | 0.00 | 57 | n.s. |
| Summer | f | Ash | -0.33 | 37 |  |
|  | m | Ash | -0.34 | 33 |  |
|  | f | Carbohydrates | -0.21 | 37 |  |
|  | m | Carbohydrates | -0.14 | 33 |  |
|  | f | Crude fat | 0.38 | 37 |  |
|  | m | Crude fat | 0.34 | 33 |  |
|  | f | Crude fibre | -0.16 | 37 |  |
|  | m | Crude fibre | -0.22 | 33 |  |
|  | f | Crude protein | 0.01 | 37 | n.s. |
|  | m | Crude protein | 0.02 | 33 | n.s. |
| Autumn | f | Ash | -0.18 | 61 |  |
|  | m | Ash | -0.19 | 6 | n≤7 |
|  | f | Carbohydrates | -0.19 | 61 |  |
|  | m | Carbohydrates | -0.08 | 6 | n≤7 |
|  | f | Crude fat | 0.26 | 62 |  |
|  | m | Crude fat | 0.28 | 6 | n≤7 |
|  | f | Crude fibre | -0.26 | 62 |  |
|  | m | Crude fibre | -0.22 | 6 | n≤7 |
|  | f | Crude protein | 0.12 | 62 |  |
|  | m | Crude protein | 0.03 | 6 | n≤7 |

| Timeframe | Sex | Fatty acid | ɛ | n | Confinement |
| --- | --- | --- | --- | --- | --- |
| Whole study period | f | FA 14:0 | -0.31 | 145 |  |
|  | m | FA 14:0 | -0.40 | 124 |  |
|  | f | FA 16:0 | -0.18 | 145 |  |
|  | m | FA 16:0 | -0.16 | 124 |  |
|  | f | FA 16:1 | -0.34 | 145 |  |
|  | m | FA 16:1 | -0.37 | 124 |  |
|  | f | FA 18:0 | 0.34 | 145 |  |
|  | m | FA 18:0 | 0.27 | 124 |  |
|  | f | FA 18:1 | 0.21 | 145 |  |
|  | m | FA 18:1 | 0.24 | 124 |  |
|  | f | LA | -0.30 | 145 |  |
|  | m | LA | -0.12 | 124 |  |
|  | f | ALA | -0.36 | 145 |  |
|  | m | ALA | -0.28 | 124 |  |
| Winter | f | FA 14:0 | 0.13 | 15 | n.s. |
|  | m | FA 14:0 | 0.04 | 28 | n.s. |
|  | f | FA 16:0 | -0.06 | 15 | n.s. |
|  | m | FA 16:0 | -0.07 | 28 | n.s. |
|  | f | FA 16:1 | -0.76 | 15 |  |
|  | m | FA 16:1 | -0.64 | 28 |  |
|  | f | FA 18:0 | 0.39 | 15 |  |
|  | m | FA 18:0 | 0.29 | 28 |  |
|  | f | FA 18:1 | 0.17 | 15 |  |
|  | m | FA 18:1 | 0.23 | 28 |  |
|  | f | LA | -0.35 | 15 |  |
|  | m | LA | -0.22 | 28 |  |
|  | f | ALA | -0.56 | 15 |  |
|  | m | ALA | -0.51 | 28 |  |
| Spring | f | FA 14:0 | -0.60 | 28 |  |
|  | m | FA 14:0 | -0.47 | 57 |  |
|  | f | FA 16:0 | -0.12 | 28 |  |
|  | m | FA 16:0 | -0.21 | 57 |  |
|  | f | FA 16:1 | -0.29 | 28 |  |
|  | m | FA 16:1 | -0.24 | 57 |  |
|  | f | FA 18:0 | 0.21 | 28 |  |
|  | m | FA 18:0 | 0.31 | 57 |  |
|  | f | FA 18:1 | 0.17 | 28 |  |
|  | m | FA 18:1 | 0.24 | 57 |  |
|  | f | LA | -0.06 | 28 |  |
|  | m | LA | -0.15 | 57 |  |
|  | f | ALA | 0.00 | 28 | n.s. |
|  | m | ALA | -0.21 | 57 |  |
| Summer | f | FA 14:0 | -0.62 | 40 |  |
|  | m | FA 14:0 | -0.68 | 33 |  |
|  | f | FA 16:0 | -0.10 | 40 |  |
|  | m | FA 16:0 | -0.12 | 33 |  |
|  | f | FA 16:1 | -0.36 | 40 |  |
|  | m | FA 16:1 | -0.40 | 33 |  |
|  | f | FA 18:0 | 0.24 | 40 |  |
|  | m | FA 18:0 | 0.18 | 33 |  |
|  | f | FA 18:1 | 0.14 | 40 |  |
|  | m | FA 18:1 | 0.20 | 33 |  |
|  | f | LA | -0.01 | 40 | n.s. |
|  | m | LA | 0.08 | 33 | n.s. |
|  | f | ALA | -0.02 | 40 | n.s. |
|  | m | ALA | -0.14 | 33 |  |
| Autumn | f | FA 14:0 | -0.08 | 62 |  |
|  | m | FA 14:0 | -0.14 | 6 | n.s. |
|  | f | FA 16:0 | -0.29 | 62 |  |
|  | m | FA 16:0 | -0.27 | 6 | n≤7 |
|  | f | FA 16:1 | -0.24 | 62 |  |
|  | m | FA 16:1 | -0.10 | 6 | n.s. |
|  | f | FA 18:0 | 0.45 | 62 |  |
|  | m | FA 18:0 | 0.31 | 6 | n≤7 |
|  | f | FA 18:1 | 0.27 | 62 |  |
|  | m | FA 18:1 | 0.40 | 6 | n≤7 |
|  | f | LA | -0.57 | 62 |  |
|  | m | LA | -0.45 | 6 | n≤7 |
|  | f | ALA | -0.70 | 62 |  |
|  | m | ALA | -0.71 | 6 | n≤7 |

| Timeframe | Age | Dry matter | ɛ | n | Confinement |
| --- | --- | --- | --- | --- | --- |
| Whole study period | a | Ash | -0.16 | 172 |  |
|  | s | Ash | -0.29 | 89 |  |
|  | a | Carbohydrates | -0.18 | 172 |  |
|  | s | Carbohydrates | -0.13 | 89 |  |
|  | a | Crude fat | 0.25 | 172 |  |
|  | s | Crude fat | 0.31 | 89 |  |
|  | a | Crude fibre | -0.23 | 172 |  |
|  | s | Crude fibre | -0.18 | 89 |  |
|  | a | Crude protein | 0.07 | 172 |  |
|  | s | Crude protein | 0.03 | 89 | n.s. |
| Winter | a | Ash | -0.01 | 35 | n.s. |
|  | s | Ash | -0.22 | 5 | n≤7 |
|  | a | Carbohydrates | -0.30 | 35 |  |
|  | s | Carbohydrates | 0.02 | 5 | n≤7 |
|  | a | Crude fat | 0.13 | 35 |  |
|  | s | Crude fat | 0.12 | 5 | n≤7 |
|  | a | Crude fibre | -0.25 | 35 |  |
|  | s | Crude fibre | -0.05 | 5 | n≤7 |
|  | a | Crude protein | 0.15 | 35 |  |
|  | s | Crude protein | 0.00 | 5 | n≤7 |
| Spring | a | Ash | -0.15 | 62 |  |
|  | s | Ash | -0.24 | 22 |  |
|  | a | Carbohydrates | -0.07 | 62 |  |
|  | s | Carbohydrates | -0.11 | 22 |  |
|  | a | Crude fat | 0.26 | 62 |  |
|  | s | Crude fat | 0.34 | 22 |  |
|  | a | Crude fibre | -0.19 | 62 |  |
|  | s | Crude fibre | -0.19 | 22 |  |
|  | a | Crude protein | 0.01 | 62 | n.s. |
|  | s | Crude protein | -0.01 | 22 | n.s. |
| Summer | a | Ash | -0.35 | 38 |  |
|  | s | Ash | -0.33 | 32 |  |
|  | a | Carbohydrates | -0.15 | 38 |  |
|  | s | Carbohydrates | -0.2 | 32 |  |
|  | a | Crude fat | 0.35 | 38 |  |
|  | s | Crude fat | 0.37 | 32 |  |
|  | a | Crude fibre | -0.20 | 38 |  |
|  | s | Crude fibre | -0.18 | 32 |  |
|  | a | Crude protein | 0.02 | 38 | n.s. |
|  | s | Crude protein | 0.01 | 32 | n.s. |
| Autumn | a | Ash | -0.09 | 37 |  |
|  | s | Ash | -0.29 | 30 |  |
|  | a | Carbohydrates | -0.25 | 37 |  |
|  | s | Carbohydrates | -0.08 | 30 |  |
|  | a | Crude fat | 0.25 | 37 |  |
|  | s | Crude fat | 0.26 | 30 |  |
|  | a | Crude fibre | -0.31 | 37 |  |
|  | s | Crude fibre | -0.19 | 30 |  |
|  | a | Crude protein | 0.13 | 37 |  |
|  | s | Crude protein | 0.08 | 30 |  |

| Timeframe | Age | Fatty acid | ɛ | n | Confinement |
| --- | --- | --- | --- | --- | --- |
| Whole study period | a | FA 14:0 | -0.30 | 176 |  |
|  | s | FA 14:0 | -0.43 | 91 |  |
|  | a | FA 16:0 | -0.16 | 176 |  |
|  | s | FA 16:0 | -0.18 | 91 |  |
|  | a | FA 16:1 | -0.40 | 176 |  |
|  | s | FA 16:1 | -0.25 | 91 |  |
|  | a | FA 18:0 | 0.31 | 176 |  |
|  | s | FA 18:0 | 0.30 | 91 |  |
|  | a | FA 18:1 | 0.23 | 176 |  |
|  | s | FA 18:1 | 0.20 | 91 |  |
|  | a | LA | -0.22 | 176 |  |
|  | s | LA | -0.20 | 91 |  |
|  | a | ALA | -0.35 | 176 |  |
|  | s | ALA | -0.27 | 91 |  |
| Winter | a | FA 14:0 | 0.09 | 37 | n.s. |
|  | s | FA 14:0 | -0.02 | 6 | n.s. |
|  | a | FA 16:0 | -0.06 | 37 | n.s. |
|  | s | FA 16:0 | -0.12 | 6 | n.s. |
|  | a | FA 16:1 | -0.68 | 37 |  |
|  | s | FA 16:1 | -0.72 | 6 | n≤7 |
|  | a | FA 18:0 | 0.33 | 37 |  |
|  | s | FA 18:0 | 0.26 | 6 | n≤7 |
|  | a | FA 18:1 | 0.19 | 37 |  |
|  | s | FA 18:1 | 0.32 | 6 | n≤7 |
|  | a | LA | -0.27 | 37 |  |
|  | s | LA | -0.23 | 6 | n≤7 |
|  | a | ALA | -0.54 | 37 |  |
|  | s | ALA | -0.46 | 6 | n≤7 |
| Spring | a | FA 14:0 | -0.51 | 62 |  |
|  | s | FA 14:0 | -0.52 | 22 |  |
|  | a | FA 16:0 | -0.19 | 62 |  |
|  | s | FA 16:0 | -0.15 | 22 |  |
|  | a | FA 16:1 | -0.27 | 62 |  |
|  | s | FA 16:1 | -0.24 | 22 |  |
|  | a | FA 18:0 | 0.29 | 62 |  |
|  | s | FA 18:0 | 0.25 | 22 |  |
|  | a | FA 18:1 | 0.25 | 62 |  |
|  | s | FA 18:1 | 0.14 | 22 | n.s. |
|  | a | LA | -0.12 | 62 |  |
|  | s | LA | -0.11 | 22 |  |
|  | a | ALA | -0.18 | 62 |  |
|  | s | ALA | -0.03 | 22 | n.s. |
| Summer | a | FA 14:0 | -0.64 | 40 |  |
|  | s | FA 14:0 | -0.66 | 33 |  |
|  | a | FA 16:0 | -0.11 | 40 |  |
|  | s | FA 16:0 | -0.11 | 33 |  |
|  | a | FA 16:1 | -0.48 | 40 |  |
|  | s | FA 16:1 | -0.26 | 33 |  |
|  | a | FA 18:0 | 0.20 | 40 |  |
|  | s | FA 18:0 | 0.22 | 33 |  |
|  | a | FA 18:1 | 0.23 | 40 |  |
|  | s | FA 18:1 | 0.09 | 33 | n.s. |
|  | a | LA | 0.02 | 40 | n.s. |
|  | s | LA | 0.04 | 33 | n.s. |
|  | a | ALA | -0.12 | 40 |  |
|  | s | ALA | -0.02 | 33 | n.s. |
| Autumn | a | FA 14:0 | 0.03 | 37 | n.s. |
|  | s | FA 14:0 | -0.21 | 30 |  |
|  | a | FA 16:0 | -0.27 | 37 |  |
|  | s | FA 16:0 | -0.31 | 30 |  |
|  | a | FA 16:1 | -0.28 | 37 |  |
|  | s | FA 16:1 | -0.17 | 30 |  |
|  | a | FA 18:0 | 0.44 | 37 |  |
|  | s | FA 18:0 | 0.42 | 30 |  |
|  | a | FA 18:1 | 0.24 | 37 |  |
|  | s | FA 18:1 | 0.33 | 30 |  |
|  | a | LA | -0.60 | 37 |  |
|  | s | LA | -0.51 | 30 |  |
|  | a | ALA | -0.70 | 37 |  |
|  | s | ALA | -0.70 | 30 |  |
