# Supplementary material for: The European Hare (Lepus europaeus): A Picky Herbivore Searching for Plant Parts Rich in Fat
Source: PLoS One. 2015 Jul 31;10(7):e0134278. doi: 10.1371/journal.pone.0134278 (PMC4521881; doi:10.1371/journal.pone.0134278)
Supplement: S2 Table — (DOC) [file pone.0134278.s002.doc]

**S2**

Model averaged coefficients for the response variables (a) ash, (b) carbohydrates, (c) crude fat, (d) crude fibre, (e) crude protein, (f) FA 14:0, (g) FA 16:0, (h) FA 16:1, (i) FA 18:0 and (j) FA 18:1 (DM: n=263; FA: n=269). The intercept stands for the estimate for female adults in summer.

| a) | Estimate | Std. Error | z value | *p* |
| --- | --- | --- | --- | --- |
| Intercept | -0.393 | 0.055 | 7.190 | <0.001 |
| Subadult | 0.047 | 0.063 | 0.751 | 0.453 |
| Winter | 0.313 | 0.062 | 5.059 | <0.001 |
| Spring | 0.196 | 0.058 | 3.403 | 0.001 |
| Autumn | 0.293 | 0.060 | 4.879 | <0.001 |
| Male | 0.044 | 0.049 | 0.897 | 0.370 |
| Subadult:Winter | -0.234 | 0.117 | 1.996 | 0.046 |
| Subadult:Spring | -0.104 | 0.074 | 1.404 | 0.160 |
| Subadult:Autumn | -0.246 | 0.075 | 3.286 | 0.001 |
| Subadult:Male | -0.038 | 0.067 | 0.562 | 0.574 |
| Winter:Male | 0.089 | 0.092 | 0.968 | 0.333 |
| Spring:Male | 0.139 | 0.071 | 1.948 | 0.051 |
| Autumn:Male | 0.063 | 0.109 | 0.580 | 0.562 |

| b) | Estimate | Std. Error | z value | *p* |
| --- | --- | --- | --- | --- |
| Intercept | -0.132 | 0.046 | 2.900 | 0.004 |
| Subadult | -0.074 | 0.049 | 1.512 | 0.131 |
| Winter | -0.168 | 0.049 | 3.418 | 0.001 |
| Spring | 0.054 | 0.044 | 1.219 | 0.223 |
| Autumn | -0.117 | 0.047 | 2.472 | 0.013 |
| Male | 0.044 | 0.035 | 1.258 | 0.208 |
| Subadult:Winter | 0.401 | 0.106 | 3.794 | 0.000 |
| Subadult:Spring | 0.019 | 0.067 | 0.280 | 0.779 |
| Subadult:Autumn | 0.231 | 0.067 | 3.428 | 0.001 |
| Subadult:Male | 0.038 | 0.058 | 0.664 | 0.507 |
| Winter:Male | -0.023 | 0.083 | 0.278 | 0.781 |
| Spring:Male | -0.093 | 0.064 | 1.442 | 0.149 |
| Autumn:Male | -0.029 | 0.097 | 0.299 | 0.765 |

| c) | Estimate | Std. Error | z value | *p* |
| --- | --- | --- | --- | --- |
| Intercept | 0.356 | 0.024 | 14.882 | <0.001 |
| Winter | -0.226 | 0.036 | 6.315 | <0.001 |
| Spring | -0.079 | 0.029 | 2.770 | 0.006 |
| Autumn | -0.108 | 0.030 | 3.601 | 0.000 |
| Subadult | 0.031 | 0.026 | 1.217 | 0.223 |
| Male | -0.012 | 0.027 | 0.443 | 0.658 |
| Subadult:Male | -0.009 | 0.046 | 0.192 | 0.848 |
| Subadult:Winter | -0.033 | 0.090 | 0.374 | 0.709 |
| Subadult:Spring | 0.057 | 0.058 | 0.986 | 0.324 |
| Subadult:Autumn | -0.006 | 0.058 | 0.108 | 0.914 |
| Winter:Male | 0.082 | 0.069 | 1.197 | 0.231 |
| Spring:Male | 0.012 | 0.056 | 0.216 | 0.829 |
| Autumn:Male | 0.055 | 0.082 | 0.674 | 0.500 |

| d) | Estimate | Std. Error | z value | *p* |
| --- | --- | --- | --- | --- |
| Intercept | -0.186 | 0.051 | 3.640 | 0.000 |
| Subadult | 0.008 | 0.049 | 0.163 | 0.871 |
| Winter | -0.008 | 0.051 | 0.156 | 0.876 |
| Spring | -0.009 | 0.039 | 0.235 | 0.814 |
| Autumn | -0.113 | 0.049 | 2.294 | 0.022 |
| Subadult:Winter | 0.219 | 0.109 | 2.000 | 0.045 |
| Subadult:Spring | -0.011 | 0.070 | 0.163 | 0.871 |
| Subadult:Autumn | 0.118 | 0.070 | 1.682 | 0.093 |
| Male | -0.016 | 0.035 | 0.464 | 0.643 |
| Subadult:Male | 0.003 | 0.064 | 0.050 | 0.960 |
| Winter:Male | 0.048 | 0.090 | 0.535 | 0.593 |
| Spring:Male | 0.010 | 0.068 | 0.151 | 0.880 |
| Autumn:Male | 0.097 | 0.104 | 0.936 | 0.349 |

| e) | Estimate | Std. Error | z value | *p* |
| --- | --- | --- | --- | --- |
| Intercept | 0.025 | 0.032 | 0.794 | 0.427 |
| Winter | 0.102 | 0.040 | 2.524 | 0.012 |
| Spring | -0.003 | 0.032 | 0.091 | 0.927 |
| Autumn | 0.095 | 0.034 | 2.801 | 0.005 |
| Male | -0.033 | 0.029 | 1.139 | 0.255 |
| Subadult | -0.023 | 0.032 | 0.713 | 0.476 |
| Subadult:Male | -0.005 | 0.049 | 0.102 | 0.919 |
| Subadult:Winter | -0.165 | 0.097 | 1.705 | 0.088 |
| Subadult:Spring | -0.012 | 0.061 | 0.197 | 0.844 |
| Subadult:Autumn | -0.050 | 0.061 | 0.811 | 0.417 |
| Winter:Male | -0.057 | 0.075 | 0.770 | 0.441 |
| Spring:Male | -0.033 | 0.059 | 0.552 | 0.581 |
| Autumn:Male | -0.090 | 0.088 | 1.028 | 0.304 |

| f) | Estimate | Std. Error | z value | *p* |
| --- | --- | --- | --- | --- |
| Intercept | -0.597 | 0.072 | 8.330 | <0.001 |
| Subadult | -0.049 | 0.065 | 0.752 | 0.452 |
| Winter | 0.654 | 0.077 | 8.446 | <0.001 |
| Spring | 0.083 | 0.075 | 1.111 | 0.266 |
| Autumn | 0.626 | 0.073 | 8.536 | <0.001 |
| Subadult:Winter | -0.088 | 0.147 | 0.601 | 0.548 |
| Subadult:Spring | 0.035 | 0.091 | 0.390 | 0.697 |
| Subadult:Autumn | -0.204 | 0.092 | 2.228 | 0.026 |
| Male | -0.006 | 0.061 | 0.101 | 0.919 |
| Winter:Male | -0.081 | 0.113 | 0.719 | 0.472 |
| Spring:Male | 0.169 | 0.088 | 1.922 | 0.055 |
| Autumn:Male | 0.011 | 0.137 | 0.079 | 0.937 |
| Subadult:Male | 0.014 | 0.092 | 0.154 | 0.878 |

| g) | Estimate | Std. Error | z value | *p* |
| --- | --- | --- | --- | --- |
| Intercept | -0.091 | 0.025 | 3.598 | 0.000 |
| Winter | 0.059 | 0.037 | 1.616 | 0.106 |
| Spring | -0.061 | 0.031 | 1.958 | 0.050 |
| Autumn | -0.192 | 0.032 | 6.077 | <0.001 |
| Male | -0.045 | 0.028 | 1.598 | 0.110 |
| Subadult | -0.007 | 0.033 | 0.220 | 0.826 |
| Subadult:Male | 0.060 | 0.045 | 1.332 | 0.183 |
| Winter:Male | 0.005 | 0.068 | 0.077 | 0.938 |
| Spring:Male | -0.059 | 0.055 | 1.080 | 0.280 |
| Autumn:Male | 0.047 | 0.081 | 0.578 | 0.563 |
| Subadult:Winter | -0.150 | 0.089 | 1.690 | 0.091 |
| Subadult:Spring | 0.010 | 0.057 | 0.183 | 0.855 |
| Subadult:Autumn | -0.047 | 0.057 | 0.818 | 0.414 |

| h) | Estimate | Std. Error | z value | *p* |
| --- | --- | --- | --- | --- |
| Intercept | -0.483 | 0.134 | 3.598 | <0.001 |
| Winter | -0.108 | 0.082 | 1.320 | 0.187 |
| Spring | 0.147 | 0.061 | 2.432 | 0.015 |
| Autumn | 0.157 | 0.060 | 2.595 | 0.009 |
| Male | 0.059 | 0.062 | 0.946 | 0.344 |
| Subadult | 0.068 | 0.056 | 1.200 | 0.230 |
| Subadult:Male | -0.073 | 0.084 | 0.870 | 0.384 |
| Winter:Male | 0.190 | 0.127 | 1.495 | 0.135 |
| Spring:Male | 0.073 | 0.103 | 0.715 | 0.475 |
| Autumn:Male | 0.218 | 0.152 | 1.434 | 0.152 |
| Subadult:Winter | -0.123 | 0.170 | 0.726 | 0.468 |
| Subadult:Spring | -0.159 | 0.107 | 1.487 | 0.137 |
| Subadult:Autumn | -0.049 | 0.108 | 0.453 | 0.651 |

| i) | Estimate | Std. Error | z value | *p* |
| --- | --- | --- | --- | --- |
| Intercept | 0.236 | 0.038 | 6.144 | <0.001 |
| Winter | 0.135 | 0.066 | 2.044 | 0.041 |
| Spring | 0.010 | 0.070 | 0.146 | 0.884 |
| Autumn | 0.205 | 0.045 | 4.557 | <0.001 |
| Male | -0.062 | 0.054 | 1.138 | 0.255 |
| Winter:Male | -0.056 | 0.093 | 0.600 | 0.549 |
| Spring:Male | 0.166 | 0.075 | 2.220 | 0.026 |
| Autumn:Male | -0.064 | 0.111 | 0.579 | 0.563 |
| Subadult | -0.018 | 0.038 | 0.482 | 0.630 |
| Subadult:Male | 0.049 | 0.064 | 0.760 | 0.447 |
| Subadult:Winter | -0.152 | 0.131 | 1.164 | 0.245 |
| Subadult:Spring | -0.057 | 0.078 | 0.736 | 0.462 |
| Subadult:Autumn | -0.027 | 0.081 | 0.332 | 0.740 |

| j) | Estimate | Std. Error | z value | *p* |
| --- | --- | --- | --- | --- |
| Intercept | 0.071 | 0.111 | 0.638 | 0.523 |
| Winter | 0.087 | 0.071 | 1.229 | 0.219 |
| Spring | 0.120 | 0.054 | 2.239 | 0.025 |
| Autumn | 0.164 | 0.073 | 2.259 | 0.024 |
| Male | 0.050 | 0.042 | 1.169 | 0.243 |
| Subadult | -0.083 | 0.077 | 1.071 | 0.284 |
| Subadult:Winter | 0.271 | 0.146 | 1.862 | 0.063 |
| Subadult:Spring | 0.032 | 0.092 | 0.345 | 0.730 |
| Subadult:Autumn | 0.217 | 0.093 | 2.335 | 0.020 |
| Subadult:Male | 0.042 | 0.085 | 0.493 | 0.622 |
| Winter:Male | 0.057 | 0.116 | 0.490 | 0.624 |
| Spring:Male | -0.035 | 0.090 | 0.390 | 0.697 |
| Autumn:Male | 0.026 | 0.137 | 0.191 | 0.849 |
