# Supplementary material for: The European Hare (Lepus europaeus): A Picky Herbivore Searching for Plant Parts Rich in Fat
Source: PLoS One. 2015 Jul 31;10(7):e0134278. doi: 10.1371/journal.pone.0134278 (PMC4521881; doi:10.1371/journal.pone.0134278)
Supplement: S3 Table — (DOC) [file pone.0134278.s003.doc]

**S3**

Mean DM and FA content [mg/g] for each plant taxon and plant group pooled over the three study years 2003-2005.

| Plant taxon | Crude ash | Crude fat | Crude protein | Carbohy-drates | Crude fibre | FA 14:0 | FA 16:0 | FA 16:1 | FA 18:0 | FA 18:1 | LA | ALA |
| --- | --- | --- | --- | --- | --- | --- | --- | --- | --- | --- | --- | --- |
| *Amaranthus sp.* | 262.60 | 18.90 | 230.80 | 284.90 | 202.60 | 0.04 | 3.13 | 0.02 | 0.42 | 0.94 | 4.69 | 9.66 |
| *Arrhenatherum elatius* | 93.70 | 26.70 | 143.40 | 447.00 | 289.20 | - | - | - | - | - | - | - |
| *Beta vulgaris* | 262.78 | 16.88 | 201.63 | 419.23 | 99.50 | 0.19 | 3.67 | 0.05 | 0.70 | 0.86 | 3.44 | 7.96 |
| *Bromus sp.* | 84.73 | 22.80 | 123.60 | 476.07 | 292.80 | - | - | - | - | - | - | - |
| *Dactylis glomerata* | 86.30 | 31.60 | 153.70 | 443.30 | 285.00 | - | - | - | - | - | - | - |
| *Daucus carota* | 177.60 | 20.00 | 126.10 | 470.30 | 206.00 | 0.30 | 4.32 | 0.02 | 0.21 | 0.29 | 5.39 | 9.47 |
| *Deschampsia flexuosa* | 70.30 | 29.00 | 125.90 | 453.80 | 321.10 | - | - | - | - | - | - | - |
| *Fagopyrum esculentum* | 232.00 | 21.70 | 248.40 | 320.20 | 177.70 | - | - | - | - | - | - | - |
| *Glycine max* | 99.60 | 89.85 | 187.85 | 398.10 | 224.65 | 1.69 | 20.94 | 0.39 | 7.47 | 5.44 | 13.65 | 40.27 |
| Unidentified grass | 87.99 | 29.44 | 112.33 | 483.27 | 286.95 | 0.41 | 4.36 | 0.04 | 0.50 | 1.36 | 5.94 | 16.84 |
| *Helianthus annuus* | 212.10 | 16.73 | 162.67 | 321.13 | 287.37 | 0.76 | 3.24 | 0.05 | 0.28 | 0.63 | 5.26 | 6.51 |
| *Hordeum vulgare* | 143.70 | 30.90 | 337.30 | 316.60 | 171.60 | 0.28 | 5.51 | 0.34 | 0.46 | 0.56 | 3.52 | 20.23 |
| *Juncus sp.* | 27.40 | 9.10 | 77.80 | 589.50 | 296.20 | - | - | - | - | - | - | - |
| *Lathyrus sp.* | 111.90 | 41.80 | 290.40 | 334.20 | 221.60 | - | - | - | - | - | - | - |
| *Leontodon sp.* | 146.60 | 46.60 | 133.50 | 418.10 | 255.20 | - | - | - | - | - | - | - |
| *Medicago sativa* | 118.30 | 22.00 | 238.00 | 367.80 | 253.90 | 0.13 | 4.96 | 0.04 | 0.86 | 0.30 | 3.86 | 11.85 |
| *Panicum miliaceum* | 54.50 | 32.90 | 98.70 | 654.40 | 159.60 | 0.50 | 5.39 | 0.07 | 0.80 | 3.95 | 17.52 | 4.67 |
| *Phacelia sp.* | 241.70 | 28.00 | 195.87 | 313.47 | 220.93 | 1.01 | 4.00 | 0.05 | 0.41 | 1.41 | 6.40 | 14.72 |
| *Robinia pseudoacacia* | 63.20 | 11.20 | 172.80 | 407.00 | 345.70 | - | - | - | - | - | - | - |
| *Secale cereale* | 118.33 | 36.60 | 207.63 | 431.10 | 206.40 | 0.18 | 5.97 | 0.57 | 0.49 | 0.54 | 4.84 | 24.02 |
| *Sinapis arvensis* | 254.60 | 16.03 | 212.83 | 280.25 | 236.33 | - | - | - | - | - | - | - |
| *Trifolium incarnatum/resupinatum* | 141.50 | 23.30 | 218.50 | 385.40 | 231.20 | - | - | - | - | - | - | - |
| *Trifolium pratense* | 88.30 | 19.50 | 121.10 | 404.80 | 366.30 | - | - | - | - | - | - | - |
| *Trifolium repens* | 116.00 | 20.50 | 201.90 | 410.50 | 251.20 | 0.41 | 4.45 | 0.04 | 0.76 | 0.44 | 4.16 | 10.25 |
| *Triticum aestivum* | 137.47 | 35.01 | 262.11 | 387.47 | 177.93 | 0.18 | 4.86 | 0.56 | 0.36 | 0.43 | 3.80 | 24.81 |
| *Zea mays* | 93.35 | 23.05 | 147.30 | 491.55 | 244.70 | 0.32 | 4.17 | 0.04 | 0.94 | 0.26 | 2.63 | 14.70 |

| Plant group | Crude ash | Crude fat | Crude protein | Carbohy-drates | Crude fibre | FA 14:0 | FA 16:0 | FA 16:1 | FA 18:0 | FA 18:1 | LA | ALA |
| --- | --- | --- | --- | --- | --- | --- | --- | --- | --- | --- | --- | --- |
| Cereals | 125.97 | 29.61 | 202.70 | 415.75 | 225.97 | 0.38 | 4.67 | 0.04 | 0.37 | 0.45 | 4.05 | 19.64 |
| Intertillage | 189.46 | 27.43 | 233.66 | 324.50 | 224.93 | 0.54 | 3.49 | 0.04 | 0.40 | 0.69 | 5.84 | 16.43 |
| Other field crops | 172.08 | 35.75 | 178.40 | 397.38 | 216.40 | 4.84 | 7.46 | 0.20 | 1.36 | 1.18 | 7.89 | 12.81 |
| Weeds/grasses | 100.86 | 25.62 | 116.57 | 450.86 | 306.07 | 0.89 | 5.60 | 0.07 | 0.49 | 0.69 | 7.20 | 10.68 |
